# PPAR-*α* Contributes to the Anti-Inflammatory Activity of Verbascoside in a Model of Inflammatory Bowel Disease in Mice

**DOI:** 10.1155/2010/917312

**Published:** 2010-06-30

**Authors:** Emanuela Esposito, Emanuela Mazzon, Irene Paterniti, Roberto Dal Toso, Giovanna Pressi, Rocco Caminiti, Salvatore Cuzzocrea

**Affiliations:** ^1^Department of Clinical and Experimental Medicine and Pharmacology, School of Medicine, University of Messina, 98123 Messina, Italy; ^2^IRCCS Centro Neurolesi “Bonino Pulejo”, via Provinciale Palermo, C.da Casazza, 98124 Messina, Italy; ^3^I.R.B. srl Altavilla, Vicentina, Italy; ^4^Department of Human Pathology, School of Medicine, University of Messina, 98123 Messina, Italy

## Abstract

The previous results suggest that peroxisome proliferator-activated receptor-alpha (PPAR)-*α*, an intracellular transcription factor activated by fatty acids, plays a role in control of inflammation. There is persuasive epidemiological and experimental evidence that dietary polyphenols have anti-inflammatory activity. In this regard, it has been demonstrated that verbascoside (VB) functions as intracellular radical scavenger and reduces the microscopic and macroscopic signs of experimental colitis. With the aim to characterize the role of PPAR-*α* in VB-mediated anti-inflammatory activity, we tested the efficacy of VB in an experimental model of inflammatory bowel disease induced by dinitrobenzene sulfonic acid, comparing mice lacking PPAR-*α* (PPAR-*α*KO) with wild type (WT) mice. Results indicate that VB-mediated anti-inflammatory activity is weakened in PPAR-*α*KO mice, compared to WT controls, especially in the inhibition of neutrophil infiltration, intestinal permeability and colon injury. These results indicate that PPAR-*α* can contribute to the anti-inflammatory activity of VB in inflammatory bowel disease.

## 1. Introduction

The inflammatory bowel diseases (IBDs) has a worldwide distribution, its pathogenesis is not clearly understood [1]. A major advance in the study of IBD has been the discovery and subsequent analysis of a number of models of mucosal inflammation that resemble IBD [2]. Recently, Blumberg and colleagues [3] have indicated that these models fall into four main categories (spontaneous, administration of exogenous agents, gene-targeted knockout or transgenic, and transfer of cells into immunodeficient animals) and each provides unique opportunities to discover insights into the nature of the pathogenesis of IBD. Dinitrobenzene sulfonic acid (DNBS)—induced colitis in experimental animals (e.g., mouse and rats) has proven to be a useful model of IBD, as it possesses many of the cell and humoral immunity characteristics found in human IBD [4]. 

Peroxisome proliferator-activated receptor (PPAR)-*α* is an intracellular transcription factor, activated by fatty acids, which play a role in inflammation [5]. PPARs are expressed in the intestine at various levels [6]. Recently, it has been demonstrated that PPAR-*α* is also expressed in the digestive tract mainly localized in the intestinal mucosa, in the small intestine, and in the colon [6]. In particular, it has been demonstrated that there is a higher expression of PPAR in the more differentiated colonic epithelial cells facing the intestinal lumen as compared to cells in the lower parts of the crypts [7]. 

In addition, it has been reported that PPAR-*α* activation can result in inhibition of nuclear factor (NF)-*κ*B activation and inflammatory genes expression [8]. Our recent results in diseases models of colitis and pleurisy show that mice lacking PPAR-*α* (PPAR-*α*KO) develop an increased inflammation as compared to wild type (WT) mice. Moreover, treatment with appropriate doses of PPAR-*α* agonists can inhibit inflammatory diseases development [9]. 

Phenylpropanoid glycosides (PPGs, also synonymous of phenylethanoid glycosides) are water soluble derivatives of phenylpropanoids (PPs), a large group of natural polyphenols widely distributed in the plant kingdom [10]. There is growing evidence that PPGs, like other plant polyphenols in general and PPs in particular, are powerful antioxidants either by direct scavenging of reactive oxygen and nitrogen species, or by acting as chain-breaking peroxyl radical scavengers [11]. Recently, PPs have been reported to be effective in the chemoprevention of tumors [10] as well as to have antithrombotic, wound healing, and cardio-protective actions [12]. There is now growing interest in the biotechnological approach to produce plant-derived active substances using nongenetically modified plant cell cultures [13]. Verbascoside (VB), containing a rhamnose unit bound to glucose which acts as a bridge, belongs to the extensive family of PPs, a class of plant-derived organic compounds that are biosynthesized from the amino acid phenylalanine. Recently, it has been demonstrated that VB promotes skin repair and ameliorates skin inflammation due to their ROS scavenging, antioxidant, iron chelating, and glutathione transferase (GST) activity inducing properties [14]. Moreover, we have recently demonstrated that VB from Syringa vulgaris IRBSV25/B cell cultures exerted protection on the chronic inflammatory response (colitis) caused by injection of DNBS in the rat [15]. 

With the aim to characterize the role of PPAR-*α* in VB-mediated anti-inflammatory activity, we tested the efficacy of VB in an experimental model of inflammatory bowel disease induced by DNBS, comparing PPAR-*α*KO and WT mice. Results indicate that in a chronic situation like inflammatory bowel disease, VB-mediated anti-inflammatory activity is weakened in PPAR-*α*KO mice compared to WT controls.

## 2. Materials and Methods

### 2.1. Animals

Mice (4-5 weeks old, 20–22 g) with a targeted disruption of the PPAR-*α* gene (PPAR-*α*KO) and littermate wild-type controls (PPAR-*α*WT) were purchased from Jackson Laboratories (Harlan Nossan, Italy). Mice homozygous for the Pparat^niJ^Gonz^−^targeted mutation mice are viable, fertile, and appear normal in appearance and behavior. Exon eight, encoding the ligand-binding domain, was disrupted by the insertion of a 1.14 kb neomycin resistance gene in the opposite transcriptional direction. After electroporation of the targeting construct into J1 ES cells, the ES cells were injected into C57BL/6N blastocysts. This stain was created on B6,129S4 background, and is maintained as a homozygote on a 129S4/SvJae background by brother sister matings. The animals were housed in a controlled environment and provided with standard rodent chow and water. Animal care was carried out in compliance with Italian regulations on protection of animals used for experimental and other scientific purposes (D.M. 116192) as well as with the EEC regulations (O.J. of E.C. L 358/112/18/1986).

### 2.2. Plant Cell Line

The Syringa vulgaris plant utilized by IRB to originate the cell line derives from the Botanical Garden of the University of Bologna, Bologna, Italy. The stabilized and highly selected cell line with high content of VB was obtained from dissected young Syringa vulgaris leaves sterilized by NaOCl and Tween 20. The stabilized and selected cell line was deposited at the Plant Cell Bank (DSMZ, Deutsche Sammlung Von Mikroorganismen und Zellkulturen, Braunschweig, Germany) coded internally IRBSV25/B and internationally DSM 16857. The IRBSV25/B plant cell line was used for the industrial culture fermentors.

### 2.3. VB Containing Extract Preparation

Syringa vulgaris IRBSV25/B cell cultures obtained at the end of the fermentation process were collected and mechanically homogenized by Ultraturrax. The solid residue, mainly cellular debris, was then separated from the aqueous phase containing VB by centrifugation at 1.000× g for 10 minutes. The yield of VB was approx. 3 g/L of the plant cell culture liquid suspension. The VB in the supernatant was recovered by solid phase extraction on XAD4 resin, followed by elution with 80/20 ethanol/water (V/V) mixture. Then, the eluted VB was concentrated under reduced pressure and lyophilized. The final extract, a pale yellow powder, contained VB in an amount over 80% (w/w), together with minor admixture (>10% w/w) of other caffeic acid derivatives. The further purification of VB was performed by repeated column chromatography on C18 silica gel and Sephadex LH20 and subsequent crystallization obtaining a final product with VB content above 97% (w/w). The standard raw extract with a purity of 50 ± 1% (w/w) was obtained from the 80% (w/w) cell extract by addition of maltodextrins.

### 2.4. HPLC Analysis

The analysis was performed using HPLC system (Agilent, series 1100 DAD, Hewlett-Packard) consisting of an autosampler, high pressure mixing pump, and the column C18 (2) Phenomenex 4.6 × 150 mm. The gradient system was Phase A—water/0.01 N phosphoric acid; and Phase B: acetonitryl/0.01 N phosphoric acid. 

The reference product used to set up the quantitative method has been internally prepared by IRB. The chemical structure of the product has been determined and confirmed by means of mass spectrometry (MS) and nuclear magnetic resonance (NMR) at the Department of Organic Chemistry of the University of Milan. The NMR spectra were recorded in CD3OD solvent on a Bruker Avance 400 instrument. 

The calibration curve of the verbascoside was in good linearity over the range of 20 *μ*g/mL–150 *μ*g/mL (*r* = 0,9997) and the average recoveries of verbascoside were 99.2% (*n* = 5, RSD 0.23%).

### 2.5. Experimental Groups

Mice were randomly allocated into the following groups: (i) *PPAR*-*α* *WT DNBS + vehicle *group. PPAR-*α*WT mice were subjected to DNBS-induced colitis plus administration of saline (*N* = 10); (ii) *PPAR*-*α* *KO DNBS + vehicle *group. PPAR-*α*KO mice were subjected to DNBS-induced colitis plus administration of saline (*N* = 10); (iii) *PPAR*-*α* *WT+VB *group. *PPAR*-*α* *WT *were subjected to DNBS-induced colitis and VB (2 mg/kg dissolved in saline) was given by gavage every 24 hour, starting from 3 hours after the administration of DNBS (*N* = 10); (iv) *PPAR*-*α* *KO+VB *group. *PPAR*-*α* *KO *were subjected to DNBS-induced colitis and VB (2 mg/kg dissolved in saline) was given by gavage every 24 hour, starting from 3 hours after the administration of DNBS (*N* = 10); (v) *PPAR*-*α* *WT Sham + vehicle. *PPAR-*α*WT mice were subjected to the surgical procedures as the above groups except that 50% ethanol were administered to the mice (*N* = 10); (vi) *PPAR*-*α* *KO Sham + vehicle. *PPAR-*α*KO mice were subjected to the surgical procedures as the above groups except that 50% ethanol were administered to the mice (*N* = 10), (vii) *PPAR*-*α* *WT Sham + VB *group. Identical to *PPAR*-*α* *WT Sham + saline group* except for the administration of VB group; and (viii) *PPAR*-*α* *KO Sham + VB *group. Identical to *PPAR*-*α* *KO Sham * + *saline group* except for the administration of VB.

### 2.6. Induction of Experimental Colitis

Colitis was induced with a very low dose of DNBS (4 mg per mouse) by using a modification of the method first described in rats [16]. In preliminary experiments, this dose of DNBS was found to induce reproducible colitis without mortality. Mice were anesthetized by enflurane. DNBS (4 mg in 100 *μ*l of 50% ethanol) was injected into the rectum through a catheter inserted 4.5 cm proximally to the anus. Carrier alone (100 *μ*l of 50% ethanol) was administered in control experiments. Thereafter, the animals were kept for 15 minutes in a Trendelenburg position to avoid reflux. After colitis induction, the animals were observed for 3 days. On Day 4, the animals were weighed and anaesthetized with chloral hydrate, and the abdomen was opened by a midline incision. The colon was removed, freed from surrounding tissues, opened along the antimesenteric border, rinsed, weighed, and processed for histology and immunohistochemistry. Colon damage (macroscopic damage score) was evaluated and scored by two independent observers according to the following criteria: 0, no damage; 1, localized hyperemia without ulcers; 2, linear ulcers with no significant inflammation; 3, linear ulcers with inflammation at one site; 4, two or more major sites of inflammation and ulceration extending >1 cm along the length of the colon; and 5–8, one point is added for each centimeter of ulceration beyond an initial 2 cm.

### 2.7. Light Microscopy

After fixation for 1 week at room temperature in Dietrich solution (14.25% ethanol, 1.85% formaldehyde, and 1% acetic acid), samples were dehydrated in graded ethanol and embedded in Paraplast (Sherwood Medical, Mahwah, New Jersey). Thereafter, 7-*μ*m sections were deparaffinized with xylene, stained with haematoxylin-eosin, and observed in a Dialux 22 Leitz (Wetziar, Germany) microscope. In order to have a quantitative estimation of colon damage, section (*n* = 6 for each animal) was scored by 2 independent observers blinded to the experimental protocol.

### 2.8. Myeloperoxidase Activity

At 4 days after intracolonic injection of DNBS, the colon was removed and weighed. The colon was analyzed for myeloperoxidase (MPO) activity, an indicator of polymorphonuclear leukocyte (PMN) accumulation, as previously described [17]. MPO activity was defined as the quantity of enzyme degrading 1 *μ*mol of peroxide per min at 37°C and was expressed in milliunits per gram weight of wet tissue.

### 2.9. Intestinal Permeability

On the 4th day of the experiment, mannitol 5 mg and lactulose dissolved in 0.5 ml of normal saline were gavaged. Individual mice were then immediately placed in metabolic cages to allow for collection of urine for 4 to 5 hours as previously described [18]. Urinary sugar excretion was assessed by gas-liquid chromatography.

### 2.10. Western Blot Analysis for PPAR-*α*

Nuclear extracts were prepared as previously described [19] with slight modifications. PPAR-*α* expression was quantified in nuclear fraction by Western blot analysis. The filters were blocked with 1x PBS, 5% (w/v) nonfat dried milk (PM) for 40 minutes at room temperature and subsequently probed with specific Abs against PPAR-*α* (1 : 500; Santa Cruz Biotechnology) in 1x PBS, 5% w/v nonfat dried milk, 0.1% Tween-20 (PMT) a 4°C, overnight. Membranes were incubated with peroxidase-conjugated bovine antimouse IgG secondary antibody or peroxidase-conjugated goat antirabbit IgG (1 : 2000, Jackson ImmunoResearch, West Grove, PA) for 1 hour at room temperature. To ascertain that blots were loaded with equal amounts of protein lysates, they were also incubated in the presence of the antibody against lamin A/C protein (1 : 5,000 Sigma-Aldrich Corp.).

### 2.11. Reagents

Biotin-blocking kit, biotin-conjugated goat antirabbit IgG, and avidin-biotin peroxidase complex were obtained from Vector Laboratories (Burlingame, CA, USA). Primary antinitrotyrosine antibody was purchased from Upstate Biotech (Saranac Lake, NY, USA). Primary ICAM-1 (CD54) for immunoistochemistry was purchased by Pharmingen. Reagents and secondary and nonspecific IgG antibody for immunohistochemical analysis were from Vector Laboratories InC. All other reagents and compounds used were obtained from Sigma Chemical Company.

### 2.12. Statistical Analysis

All values in the figures and text are expressed as mean ± SEM of *N* observations, where *n* represents the number of animals studied. In the experiments involving histology or immunohistochemistry, the figures shown are representative of at least 3 experiments performed on different experimental days. Data sets were examined by one- and two-way analysis of variance and individual group means were then compared with Student's unpaired *t*-test. Nonparametric data were analyzed with the Fisher's exact test. A *P*-value less than .05 was considered significant.

## 3. Results

### 3.1. Effects of VB Treatment on PPAR-*α* Expression in Colon Tissues

**Figure 1:**
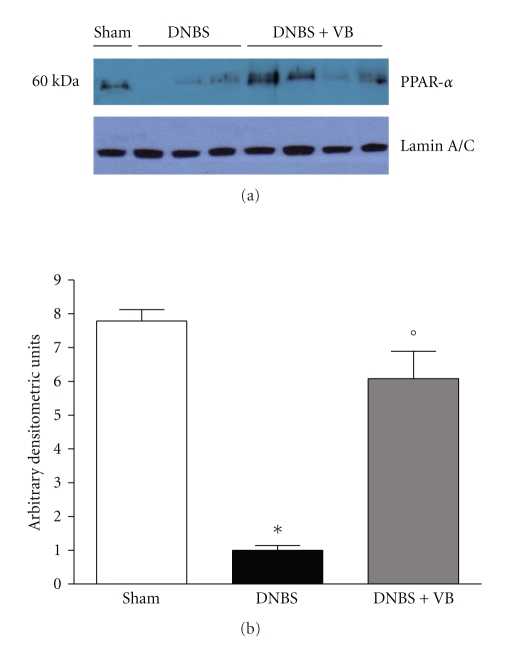
Western blot analysis for PPAR-*α* in nuclear extracts. By Western blot analysis, a basal level of PPAR-*α* expression was detected in colon tissues from sham-operated mice ((a) and (b)). Four days after DNBS administration, PPAR-*α* expression was significantly reduced. VB treatments significantly reduced the DNBS-induced inhibition of PPAR-*α* expression. The relative expression of the protein bands was standardized for densitometric analysis to lamin A/C levels and reported in (b) expressed as mean ± s.e.m. from *n* = 5/6 colon for each group. *P < .01 versus Sham; °*P* < .01 versus DNBS-WT group.

Previous studies have demonstrated an important role for PPAR-*α* in experimental colitis [15] and have hypothesized that the ability of VB to reduce inflammation may be dependent on activation of PPAR-*α*. Thus, we evaluated PPAR-*α* expression in the nuclear fractions from colon tissue by Western Blot analysis. A basal level of PPAR-*α* was detected in colon tissue from sham-treated mice, whereas PPAR-*α* levels were substantially reduced in DNBS-treated mice (Figure 1(a) and 1(b)). The effect of DNBS was significantly reduced by VB administration (Figure 1(a) and 1(b)).

### 3.2. Role of Functional PPAR-*α* Gene in the Anti-Inflammatory Activity of VB on the Degree of Colitis

**Figure 2:**
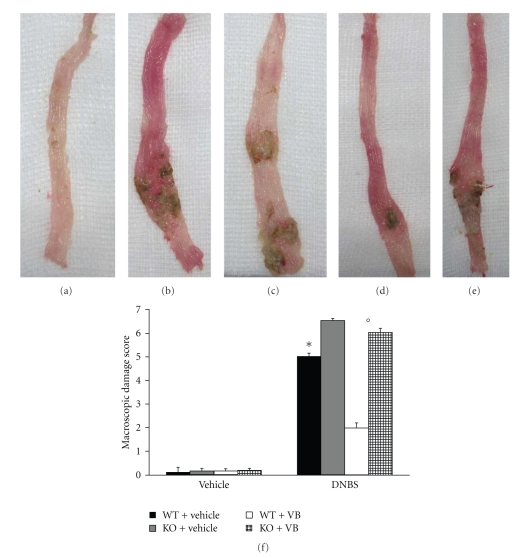
Role of PPAR-*α* in the anti-inflammatory property of VB on clinical expression of DNBS-induced colitis and macroscopic damage score. Colon tissues from sham-treated mice (a), colon tissues from DNBS-treated PPAR-*α*WT (b) or DNBS-treated PPAR-*α*KO mice (c) from mice at 4 days post DNBS administration, and the colon tissues collected from DNBS-treated mice which have received VB treatment (d). The treatment of PPAR-*α*WT with VB resulted in a significant decrease in the extent and severity of the macroscopic signs (d). The genetic absence of the PPAR-*α* receptor significantly reduced the effect of the VB treatment (e). The macroscopic damage score was made by two independent observers (f). Data are means ± SEM of 10 mice for each group. *P < .01 versus Sham; °*P* < .05 versus DNBS-WT group.

**Figure 3:**
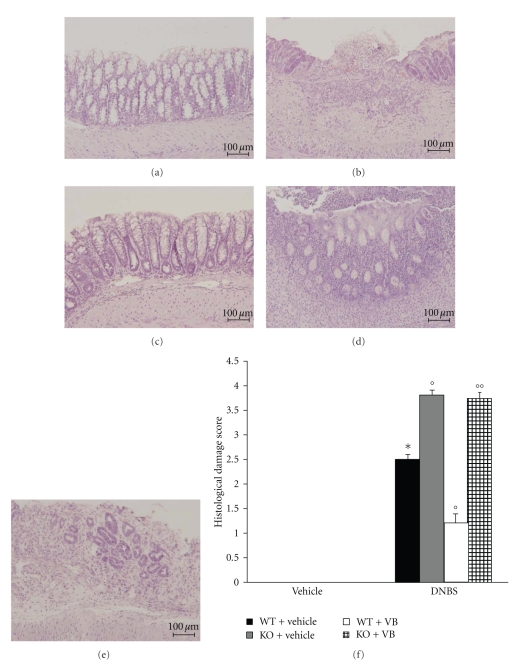
No histological alteration was observed in the colon section from sham-operated mice (a). Mucosal injury was produced after DNBS administration characterized by absence of epithelium and a massive mucosal and submucosal infiltration of inflammatory cells (b). Treatment with VB corrected the disturbances in morphology associated with DNBS administration in PPAR-*α*WT (c). The absence of PPAR-*α* gene significantly increases the extent and severity of the histological signs of colon injury (d). The genetic absence of the PPAR-*α* receptor significantly reduced the effect of the VB treatment (e). The histological score (f) was made by two independent observers. Figure 3 is representative of at least 3 experiments performed on different experimental days. Data are means ± SEM of 10 mice for each group. *P < .01 versus Sham; °*P* < .05 versus DNBS-WT group.

**Figure 4:**
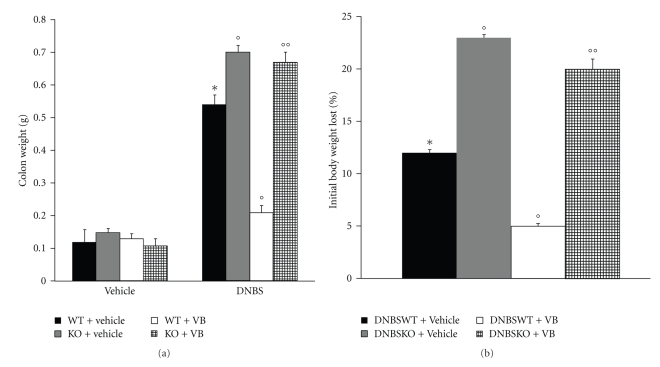
Role of PPAR-*α* in the anti-inflammatory property of VB on colon weight and body weight lost. Four days after DNBS administration a significant increase in colon weight (a) and body weight lost (b) was observed. Treatment with VB significantly reduced the increased colon weight and lowered body weight lost. The absence of PPAR-*α* gene significantly abolished the effect of VB. Figure 4 is representative of all the animals in each group. Data are means ± SEM of 10 mice for each group. *P < .01  versus Sham; °*P* < .01 versus DNBS-WT group.

Four days after intracolonic administration of DNBS, the colon appeared flaccid and filled with liquid stool. The macroscopic inspection of cecum, colon, and rectum showed presence of mucosal congestion, erosion, and hemorrhagic ulcerations (Figure 2(b), see macroscopic score Figure 2(f)). The histopathological features included a transmural necrosis and edema and a diffuse leukocyte cellular infiltrate in the submucosa of colon section from DNBS-treated PPAR-*α*WT mice (Figure 3(b), see histological score Figure 3(f)). The observed inflammatory changes of the large intestine were associated with an increase in the weight of the colon (Figure 4(a)). The absence of PPAR-*α* gene significantly increases the extent and severity of the macroscopic signs (Figure 2(c), see macroscopic score Figure 2(f)), histological indications of colon injury (Figure 3(c), see histological score Figure 3(f)), as well as the colon weight (Figure 4(a)). Four days after colitis induced by DNBS treatment, all mice had diarrhea and a significant reduction in body weight (compared with the control groups of mice). Absence of a functional PPAR-*α* gene in PPAR-*α*KO mice resulted in a significant augmentation of lost of body weight (Figure 4(b)). No macroscopic (Figure 2(a), see macroscopic score Figure 2(f) **) **or histological (Figure 3(a), see macroscopic score Figure 3(f) **) **alteration was observed in the colon tissue from vehicle-treated PPAR-*α*WT and PPAR-*α*KO mice. On the other hand, the treatment of PPAR-*α*WT with VB resulted in a significant decrease in the extent and severity of the macroscopic (Figure 2(d), see macroscopic score Figure 2(f)), histological signs of colon injury (Figure 2(d), see macroscopic score Figure 2(f)), the colon weight (Figure 3(a)), and the lost of body weight (Figure 3(b)). The genetic absence of the PPAR-*α* receptor significantly blocked the beneficial effect of VB (Figures 2 and 3, see macroscopic score Figures 2(f) and 3(f)).

### 3.3. Role of PPAR-*α* in VB-Induced Inhibition of PMN Infiltration

**Figure 5:**
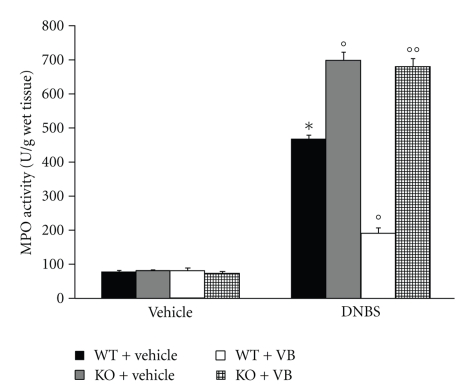
Effect of endogenous PPAR-*α* on neutrophil infiltration. Myeloperoxidase (MPO) activity was significantly increased in DNBS-treated PPAR-*α*WT mice in comparison to SHAM. MPO activity was significantly enhanced in DNBS-treated PPAR-*α*KO mice. Treatment with VB significantly reduced the colon MPO activity. The genetic absence of the PPAR-*α* receptor significantly blocked the effect of VB treatment. Data are means ± SEM of 10 mice for each group. *P < .01 versus sham; °*P* < .01 versus DNBS WT.

The colitis caused by DNBS was also characterized by an increase in myeloperoxidase activity, an indicator of the neutrophils' accumulation in the colon (Figure 5). This finding is consistent with our observation made with light microscopy that the colon from vehicle-treated DBNS-rats contained a large number of neutrophils. In PPAR-*α*KO mice, colon myeloperoxidase activity was markedly increased in comparison to those of PPAR-*α*WT animals (Figure 5). On the contrary, VB significantly reduced the degree of PMN infiltration (determined as increase in MPO activity) in inflamed colon. The genetic absence of the PPAR-*α* receptor significantly blunted the effect of the VB on the neutrophil infiltration (Figure 5).

### 3.4. Intestinal Permeability

**Figure 6:**
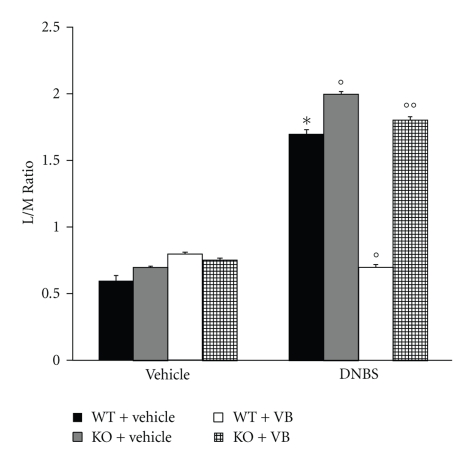
Effects of PPAR-*α* on intestinal permeability. Permeability probes (lactulose and mannitol) were administered by gavage, and urinary lactulose and mannitol excretion were measured in urine collected over a 4- to 5-hour postgavage period, and L/M ratios were calculated. The L/M ratio was unchanged in the sham-treated mice. On the contrary, at 4 days after colitis, the L/M ratios were significantly higher in the urinary samples from DNBS-treated PPAR-*α*WT mice in comparison with the sham animals. The absence of a functional PPAR-*α* gene in PPAR-*α*KO mice resulted in a more pronounced increase of L/M ratios. Treatment with VB significantly reduced the increased L/M ratios, while the absence of PPAR-*α* gene significantly abolished the effect of VB. Data are means ± SEM of 10 mice for each group. *P < .01 versus sham; °*P* < .01 DNBS WT.

For assessing baseline small bowel permeability, permeability probes (lactulose and mannitol) were administered by gavage, and urinary lactulose and mannitol excretion were measured in urine collected over a 4- to 5-hour post gavage period and L/M ratios were calculated. As indicated in Figure 6, the L/M ratios were unchanged in the sham PPAR-*α*WT and in sham PPAR-*α*KO mice. On the contrary, in the urinary samples from PPAR-*α*WT mice injected with DNBS L/M ratios were significantly higher in comparison to the sham animals. The absence of PPAR-*α* in mice (animals with the PPAR-*α*KO phenotype) resulted in a pronounced increase of L/M ratios (Figure 6). On the contrary, VB significantly reduced the increase in L/M ratios. The genetic absence of the PPAR-*α* receptor significantly blocked the effect of the VB on L/M ratios (Figure 6).

## 4. Discussion

 In the present paper, we show that the absence of PPAR-*α* in mice results in a reduced anti-inflammatory response to VB treatment in an IBD model. These results are in agreement with our previous observations indicating that PPAR-*α*KO mice are more susceptible to induction of inflammatory bowel disease [20]. 

There is growing interest in the role of complementary and alternative medicine (CAM) in health and disease. There are a myriad of CAM treatments that may prove beneficial for patients with IBD. Unfortunately, the scientific basis for the use of these modalities frequently is lacking, and safety has not been assessed. Recently, we have demonstrated that VB from Syringa vulgaris IRBSV25/B cell cultures attenuates DNBS-induced colitis in the rats, a well-established model of acute intestinal inflammation with some shared features of Crohn's disease [15]. 

Various studies have regarded polyphenols as PPAR ligands [21]. In particular they have demonstrated that genistein increases the expression of genes involved in lipid catabolism in a PPAR*α*-dependent mechanism [22]. Moreover, it has been also demonstrated that epigallocatechin-3-gallate (EGCG) binds PPAR-*α* [21]. Therefore, other studies have also demonstrated that amentoflavone upregulated PPAR-*γ* expression in A549 human lung cell as well as that curcumin dramatically induced the expression of PPAR-*γ* at levels of transcription and translation [23]. 

These findings implied a potential therapeutic value of PPARs ligands in treatment of inflammatory disease [24, 25].

PPAR-*α* itself is also able to directly mediate some anti-inflammatory effects and it has been shown that agonist-induced activation inhibits a number of inflammatory parameters including TNF-*α* production, iNOS, COX-2, and adhesion molecule expression as well cell infiltration in the tissues [5].

Based on these observations, we performed studies in the attempt to determine whether the presence and/or the stimulation of PPAR-*α* could enhance the VB anti-inflammatory efficacy. For this purpose, we used an experimental model of inflammatory bowel disease induced by DNBS using PPAR-*α*KO mice compared to WT.

VB-type phenolic ester glycosides showed various biological activities, from antibiotic and antifeedant to immunodepressive. Moreover, VB exhibits antioxidative effects [26] and antibacterial [27]. VB has been also shown to modulate nitric oxide (NO) production and the expression of inducible nitric oxide synthase (iNOS) in activated macrophages [28, 29]. It also inhibits histamine, arachidonic acid release, and prostaglandin E2 production in RBL-2H3 mast cells suggesting a possible application of the compound as anti-inflammatory remedy [30]. 

In the present study, we have clearly confirmed that the increased colon injury in the colon tissue from DNBS-treated PPAR-*α*KO mice correlated with the enhanced leukocyte infiltration, as assessed by the specific granulocyte enzyme myeloperoxidase. These observations are in agreement with a previous study [20]. Moreover, in the present study we clearly demonstrate that when WT and PPAR-*α*KO mice were treated with VB, a significant inhibition of neutrophils infiltration as well as tissue injury was observed in WT but not in PPAR-*α*KO mice. 

Furthermore, intestinal permeability reflects the integrity of the intestinal mucosal barrier, which restricts the passive permeation of luminal substances [31]. It is well known that altered intestinal permeability has been reported in various intestinal conditions associated with diarrhea such as celiac disease [32], IBD [33], infectious gastroenteritis [34–36], and food intolerance or allergy [37, 38]. It is possible to assess intestinal permeability *in vivo* by measuring the urinary excretion of hydrosoluble and not degradable probes given orally. The lactulose/mannitol (L/M) test uses a mixture of two unmetabolized sugars, minimizing the influence of variables such as gastric emptying, intestinal motility, and renal function, which affect both markers equally. Moreover, it is a nontoxic, noninvasive test and the laboratory procedure is simple and inexpensive [39]. The L/M test is used in the diagnosis and follow-up of celiac [40–42] and Crohn's disease [43, 44]. 

As clearly demonstrated in this study, the absence of PPAR-*α* in mice (animals with the PPAR-*α*KO phenotype) resulted in a pronounced increase in L/M ratios observed at 4 days after DNBS administration in comparison with PPAR-*α*WT mice. Moreover, when WT and PPAR-*α*KO mice were treated with VB, a significant inhibition in L/M ratios was observed in WT but not in PPAR-*α*KO mice. Is also important to point out that the genetic inhibition of PPAR-*α* did not change the physiological L/M ratios in the sham-operated mice.

The results here described clearly indicate that the anti-inflammatory efficacy of VB treatment is favored by the presence of PPAR-*α*. Moreover, previous studies showed that PPAR-*α* agonists exert some anti-inflammatory activity [45]. The efficacy of VB treatment in inflammatory and autoimmune diseases is an important therapeutic subject and while some patients obtain clinical benefits from treatment, others are not responsive or even resistant to therapy. Results discussed here suggest a new mechanism contributing to determine the full VB efficacy and suggest future studies aimed to analyze the possible relevance of PPAR-*α* in other human inflammatory disease models, such as sepsis.

In conclusion, our results clearly indicate that PPAR-*α* can contribute by enhancing the anti-inflammatory activity of VB in DNBS-induced inflammatory bowel disease model. These observations suggest that VB could use the same pathway of PPAR-*α* agonists in inflammatory diseases for its beneficial effects. 

## References

[1] Cuzzocrea S (2003). Emerging biotherapies for inflammatory bowel disease. *Expert Opinion on Emerging Drugs*.

[2] Matsumoto S, Okabe Y, Setoyama H (1998). Inflammatory bowel disease-like enteritis and caecitis in a senescence accelerated mouse P1/Yit strain. *Gut*.

[3] Blumberg RS, Saubermann LJ, Strober W (1999). Animal models of mucosal inflammation and their relation to human inflammatory bowel disease. *Current Opinion in Immunology*.

[4] Mazzon E, Puzzolo D, Caputi AP, Cuzzocrea S (2002). Role of IL-10 in hepatocyte tight junction alteration in mouse model of experimental colitis. *Molecular Medicine*.

[5] Neve BP, Corseaux D, Chinetti G (2001). PPAR*α* agonists inhibit tissue factor expression in human monocytes and macrophages. *Circulation*.

[6] Escher P, Braissant O, Basu-Modak S, Michalik L, Wahli W, Desvergne B (2001). Rat PPARs: quantitative analysis in adult rat tissues and regulation in fasting and refeeding. *Endocrinology*.

[7] Mansén A, Guardiola-Diaz H, Rafter J, Branting C, Gustafsson J-Å (1996). Expression of the peroxisome proliferator-activated receptor (PPAR) in the mouse colonic mucosa. *Biochemical and Biophysical Research Communications*.

[8] Okamoto H, Iwamoto T, Kotake S, Momohara S, Yamanaka H, Kamatani N (2005). Inhibition of NK-*κ*B signaling by fenofibrate, a peroxisome proliferator-activated receptor-*α* ligand, presents a therapeutic strategy for rheumatoid arthritis. *Clinical and Experimental Rheumatology*.

[9] Cuzzocrea S, Mazzon E, Di Paola R (2006). The role of the peroxisome proliferator-activated receptor-*α* (PPAR-*α*) in the regulation of acute inflammation. *Journal of Leukocyte Biology*.

[10] Korkina LG (2007). Phenylpropanoids as naturally occurring antioxidants: from plant defense to human health. *Cellular and Molecular Biology*.

[11] Afanas’ev IB (2005). Superoxide and nitric oxide in pathological conditions associated with iron overload. The effects of antioxidants and chelators. *Current Medicinal Chemistry*.

[12] Kris-Etherton PM, Hecker KD, Bonanome A (2002). Bioactive compounds in foods: their role in the prevention of cardiovascular disease and cancer. *American Journal of Medicine*.

[13] Minghetti P, Casiraghi A, Cilurzo F, Montanari L (2000). Development of local patches containing melilot extract and ex vivo-in vivo evaluation of skin permeation. *European Journal of Pharmaceutical Sciences*.

[14] Korkina LG, Mikhal’chik EV, Suprun MV, Pastore S, Dal Toso R (2007). Molecular mechanisms underlying wound healing and anti-inflammatory properties of naturally occurring biotechnologically produced phenylpropanoid glycosides. *Cellular and Molecular Biology*.

[15] Mazzon E, Esposito E, Di Paola R (2009). Effects of verbascoside biotechnologically produced by Syringa vulgaris plant cell cultures in a rodent model of colitis. *Naunyn-Schmiedeberg’s Archives of Pharmacology*.

[16] Sturiale S, Barbara G, Qiu B (1999). Neutral endopeptidase (EC 3.4.24.11) terminates colitis by degrading substance P. *Proceedings of the National Academy of Sciences of the United States of America*.

[17] Mullane KM, Kraemer R, Smith B (1985). Myeloperoxidase activity as a quantitative assessment of neutrophil infiltration into ischemic myocardium. *Journal of Pharmacological Methods*.

[18] Mazzon E, Sautebin L, Caputi AP, Cuzzocrea S (2006). 5-lipoxygenase modulates the alteration of paracellular barrier function in mice ileum during experimental colitis. *Shock*.

[19] Bethea JR, Castro M, Keane RW, Lee TT, Dietrich WD, Yezierski RP (1998). Traumatic spinal cord injury induces nuclear factor-*κ*B activation. *Journal of Neuroscience*.

[20] Cuzzocrea S, Di Paola R, Mazzon E (2004). Role of endogenous and exogenous ligands for the peroxisome proliferators activated receptors alpha (PPAR-*α*) in the development of inflammatory bowel disease in mice. *Laboratory Investigation*.

[21] Yoon J-H, Baek SJ (2005). Molecular targets of dietary polyphenols with anti-inflammatory properties. *Yonsei Medical Journal*.

[22] Kim S, Shin H-J, Kim SY (2004). Genistein enhances expression of genes involved in fatty acid catabolism through activation of PPAR*α*. *Molecular and Cellular Endocrinology*.

[23] Xu J, Fu Y, Chen A (2003). Activation of peroxisome proliferator-activated receptor-*γ* contributes to the inhibitory effects of curcumin on rat hepatic stellate cell growth. *American Journal of Physiology*.

[24] Becker J, Delayre-Orthez C, Frossard N, Pons F (2006). Regulation of inflammation by PPARs: a future approach to treat lung inflammatory diseases?. *Fundamental and Clinical Pharmacology*.

[25] Chung JH, Seo AY, Chung SW (2008). Molecular mechanism of PPAR in the regulation of age-related inflammation. *Ageing Research Reviews*.

[26] Xiong Q, Kadota S, Tani T, Namba T (1996). Antioxidative effects of phenylethanoids from Cistanche deserticola. *Biological and Pharmaceutical Bulletin*.

[27] Wong IYF, He Z-D, Huang Y, Chen Z-Y (2001). Antioxidative activities of phenylethanoid glycosides from Ligustrum purpurascens. *Journal of Agricultural and Food Chemistry*.

[28] Xiong Q, Tezuka Y, Kaneko T (2000). Inhibition of nitric oxide by phenylethanoids in activated macrophages. *European Journal of Pharmacology*.

[29] Lee JY, Woo E-R, Kang KW (2005). Inhibition of lipopolysaccharide-inducible nitric oxide synthase expression by acteoside through blocking of AP-1 activation. *Journal of Ethnopharmacology*.

[30] Lee JH, Ji YL, Hyo SK (2006). The effect of acteoside on histamine release and arachidonic acid release in RBL-2H3 mast cells. *Archives of Pharmacal Research*.

[31] Hollander D (1999). Intestinal permeability, leaky gut, and intestinal disorders. *Current Gastroenterology Reports*.

[32] Bjarnason I, Maxton D, Reynolds AP, Catt S, Peters TJ, Menzies IS (1994). Comparison of four markers of intestinal permeability in control subjects and patients with coeliac disease. *Scandinavian Journal of Gastroenterology*.

[33] Jenkins RT, Chem C, Jones DB (1987). Reversibility of increased intestinal permeability to 51Cr-EDTA in patients with gastrointestinal inflammatory diseases. *American Journal of Gastroenterology*.

[34] Zhang Y, Lee B, Thompson M (2000). Lactulose—mannitol intestinal permeability test in children with diarrhea caused by rotavirus and Cryptosporidium. Diarrhea Working Group. *Journal of Pediatric Gastroenterology and Nutrition*.

[35] Alam AN, Sarker SA, Wahed MA, Khatun M, Rahaman MM (1994). Enteric protein loss and intestinal permeability changes in children during acute shigellosis and after recovery: effect of zinc supplementation. *Gut*.

[36] Zuckerman MJ, Watts MT, Bhatt BD, Ho H (1993). Intestinal permeability to [51Cr]EDTA in infectious diarrhea. *Digestive Diseases and Sciences*.

[37] Schrander JJP, Unsalan-Hooyen RWM, Forget PP, Jansen J (1990). [51Cr]EDTA intestinal permeability in children with cow’s milk intolerance. *Journal of Pediatric Gastroenterology and Nutrition*.

[38] Ukabam SO, Mann RJ, Cooper BT (1984). Small intestinal permeability to sugars in patients with atopic eczema. *British Journal of Dermatology*.

[39] Bjarnason I, MacPherson A, Hollander D (1995). Intestinal permeability: an overview. *Gastroenterology*.

[40] Juby LD, Rothwell J, Axon ATR (1989). Lactulose/mannitol test: an ideal screen for celiac disease. *Gastroenterology*.

[41] Vogelsang H, Genser D, Wyatt J (1995). Screening for celiac disease: a prospective study on the value of noninvasive tests. *American Journal of Gastroenterology*.

[42] Hamilton I, Cobden I, Rothwell J, Axon ATR (1982). Intestinal permeability in coeliac disease: the response to gluten withdrawal and single-dose gluten challenge. *Gut*.

[43] Wyatt J, Vogelsang H, Hubl W, Waldhoer T, Lochs H (1993). Intestinal permeability and the prediction of relapse in Crohn’s disease. *Lancet*.

[44] D’Incà R, Di Leo V, Corrao G (1999). Intestinal permeability test as a predictor of clinical course in Crohn’s disease. *American Journal of Gastroenterology*.

[45] Lovett-Racke AE, Hussain RZ, Northrop S (2004). Peroxisome proliferator-activated receptor *α* agonists as therapy for autoimmune disease. *Journal of Immunology*.

